# Artificial Intelligence Based Optimization for the Se(IV) Removal from Aqueous Solution by Reduced Graphene Oxide-Supported Nanoscale Zero-Valent Iron Composites

**DOI:** 10.3390/ma11030428

**Published:** 2018-03-15

**Authors:** Rensheng Cao, Mingyi Fan, Jiwei Hu, Wenqian Ruan, Xianliang Wu, Xionghui Wei

**Affiliations:** 1 Guizhou Provincial Key Laboratory for Information Systems of Mountainous Areas and Protection of Ecological Environment, Guizhou Normal University, Guiyang 550001, China; 18230825324@163.com (R.C.); fanmingyifmy@163.com (M.F.); wenqianruan@yahoo.com (W.R.); 2 Cultivation Base of Guizhou National Key Laboratory of Mountainous Karst Eco-environment, Guizhou Normal University, Guiyang 550001, China; wuxianliang1995@163.com; 3 Department of Applied Chemistry, College of Chemistry and Molecular Engineering, Peking University, Beijing 100871, China; xhwei@pku.edu.cn

**Keywords:** Se(IV), artificial intelligence, artificial neural networks, genetic algorithm, particle swarm optimization

## Abstract

Highly promising artificial intelligence tools, including neural network (ANN), genetic algorithm (GA) and particle swarm optimization (PSO), were applied in the present study to develop an approach for the evaluation of Se(IV) removal from aqueous solutions by reduced graphene oxide-supported nanoscale zero-valent iron (nZVI/rGO) composites. Both GA and PSO were used to optimize the parameters of ANN. The effect of operational parameters (i.e., initial pH, temperature, contact time and initial Se(IV) concentration) on the removal efficiency was examined using response surface methodology (RSM), which was also utilized to obtain a dataset for the ANN training. The ANN-GA model results (with a prediction error of 2.88%) showed a better agreement with the experimental data than the ANN-PSO model results (with a prediction error of 4.63%) and the RSM model results (with a prediction error of 5.56%), thus the ANN-GA model was an ideal choice for modeling and optimizing the Se(IV) removal by the nZVI/rGO composites due to its low prediction error. The analysis of the experimental data illustrates that the removal process of Se(IV) obeyed the Langmuir isotherm and the pseudo-second-order kinetic model. Furthermore, the Se 3d and 3p peaks found in XPS spectra for the nZVI/rGO composites after removing treatment illustrates that the removal of Se(IV) was mainly through the adsorption and reduction mechanisms.

## 1. Introduction

Selenium (Se) is a metalloid that can exist in a variety of valence states, including selenide (Se(-II)), elemental Se (Se(0)), selenite (Se(IV)) and selenate (Se(VI)) [1,2]. As a valuable element, Se can be widely used in photosensitive drums for copying machines, photoelectric devices, pigments, metallurgical additives, glass manufacturing and semi-conductors [3,4]. Its increasing usage has generated a considerable amount of selenium-contaminated wastewater, mainly containing selenate and selenite [5]. Although Se is an essential trace element for human health at the concentration range from 0.8 to 1.7 µmol/L, excessive intake of Se could lead to serious health issues [6,7]. The United States Environmental Protection Agency (US EPA) has mandated the maximum contaminant level of Se in drinking water at 50 µg/L [5]. The acute toxicity of Se(IV) is almost 10 times higher than that of Se(VI) and both species generally exist simultaneously in aerobic surface water. Therefore, there is an urgent need for developing efficient and feasible methods to remove Se(IV) from waste water [1].

Using the green nanoparticles with a low cost and high adsorption capacity is a good approach to remove pollutants [8]. Nanoscale zero-valent iron (NZVI) particles have been considered as one of the most promising permeable barrier materials applied in wastewater treatment because of their extremely small particle size, large specific surface area, excellent in-situ reactivity and high injectability into aqueous solutions [9,10,11]. Previous reports have indicated that nanoscale zero-valent iron (NZVI) can remove a range of environmental contaminants, such as chlorinated solvents, organochlorine pesticides, polychlorinated biphenyls, organic dyes, and inorganic pollutants, tetracycline [12,13]. However, some technical issues, e.g., easy agglomeration, poor stability, shortage of durability and mechanical strength [14,15], prevent the practical application of NZVI particles. Furthermore, the aggregation is hard to be avoided due to the magnetic interaction among the NZVI particles [8]. Previous researchers have shown that the aggregation of NZVI particles could be reduced when they were supported on the substrates. In this way, their dispersion ability and specific surface area were improved and their reactivity was also enhanced [16,17]. 

Reduced graphene oxide (rGO) has recently received an intensive attention due to its exceptional electron transport and mechanical properties as well as high surface area [12,18,19]. And the synthesis of most rGO-based multifunctional composites stems from graphene oxide (GO) [20,21], which can be readily made from low-cost natural graphite in a large scale. GO and rGO were broadly used in the adsorption processes to remove the pollutants from wastewater [22], such as heavy metal ions [23,24], radionuclides [25], and many cationic or anionic dyes [26]. NZVI particles supported on graphene do not only increase the dispersion ability and stability but also strengthen electron transfer and removal of pollutants by coupling the advantages of NZVI reactivity with graphene adsorption ability [8]. Therefore, the composites based on rGO are significantly more applicable than those based on bare nanomaterials [12].

The most important stage in a removal process is to improve a system and increase the efficiency of the process via modeling and optimization without increasing the costs [27]. Because the removal processes involve interactions of variables and non-linear behavior, it is highly important that optimum experimental conditions are determined to obtain a maximum efficiency. Optimization techniques based on artificial intelligence (AI) approaches can be an effective solution in these processes [28]. AI tools have broadly applied in various fields, e.g., image understanding, intelligent internet search, autonomous driving, pattern recognition, automatic programming, robotics, human-computer games and big data. As one of the AI primary tools, artificial neural network (ANN) inspired by biological neurons can well describe multivariate nonlinear problems with the suitable amount of data and the appropriate training algorithm applied [29]. Optimization algorithms, e.g., genetic algorithm (GA) and particle swarm optimization (PSO), were inspired from social behavior or natural phenomena, which can be utilized to solve complex problems [30]. Both ANN and GA play an important role in various fields of science and technology. Based on the principles of evolution through a natural selection, GA has been found to be suitable for finding the optimum solutions in some complex systems [31]. PSO is a stochastic optimization technique motivated by the behavior of a bird’s flock [32]. 

The objective of this study was to apply the reduced graphene oxide-supported nanoscale zero-valent iron (nZVI/rGO) composites in Se(IV) removal from aqueous solutions. Response surface methodology (RSM) as an experimental design tool was employed to examine the effect of process parameters (viz. temperature, contact time, initial pH and initial Se(IV) concentration) on removal efficiency, which was combined with AI techniques (including ANN-PSO and ANN-GA) applied for modeling the removal process of Se(IV) and predicting its maximum removal efficiency. Furthermore, the experimental data obtained were fitted to the adsorption isotherms (Langmuir, Freundlich and Redlich Peterson) and the removal kinetic models (pseudo-first-order, pseudo-second-order and intraparticle diffusion). Finally, the thermodynamic study and the XPS analysis were carried out to explore the mechanisms of Se(IV) removal process.

## 2. Materials and Methods

All commonly used chemicals of analytical grades (i.e., HCl, NaOH, Na_2_SeO_3_, FeSO_4_·7H_2_O, and NaBH_4_) were applied without further purification in this study, and graphite powder (purity > 99.85%, particle size < 30 μm) was obtained from Sinopharm Chemical Reagent (Beijing, China). The Se(IV) stock solution (1000 mg/L) was prepared by dissolving an amount of Na_2_SeO_3_ in deionized water. Graphene oxide was synthesized using the modified Hummers method [33], and nZVI/rGO magnetic composites were synthesized according to our previous reports [34,35].

### 2.1. Characterization of nZVI/rGO Composites

The characterization (XRD, SEM, TEM, Raman, FTIR, and N_2_ sorption) of nZVI/rGO composites was carried out in our earlier study [34]. The *X*-ray photoelectron spectroscopy (XPS) measurements were recorded on an ESCALAB 250Xi spectrometer using monochromatized Al Kα radiation (hν = 1486.6 eV), and all binding energies were corrected with the binding energy of C1s (hν = 284.8 eV) as a reference.

### 2.2. Adsorption Experiments

Removal of the Se(IV) solutions by nZVI/rGO composites was performed in a 100 mL conical flask. 30 mg of the nZVI/rGO composites was added to 50 mL of Se(IV) solution, which was shaken in a vibrator (HZQ-F160, HDL, Jingda Instrument Manufacturing Co. Ltd, Jiangsu, China) at 200 rpm. Batch removal experiments of Se(IV) (single factor experiments) were carried out to examine the effect of initial Se(IV) concentration (C), contact time (t), initial pH and operating temperature (T) on the removal efficiency of Se(IV), and the initial pH of Se(IV) solution was adjusted to the desired value by using 0.1 mol/L HCl or 0.1 mol/L NaOH. The nZVI/rGO composites were separated from aqueous solutions by a magnet after the removal experiment. The Se(IV) concentration in the sample solutions was determined through an inductively coupled plasma-optical emission spectroscopy (optima 5300 V, Perkin Elmer Corporation, Waltham, MA, USA). All experiments were carried out in triplicates and the average values of the results obtained were used for data analysis. 

The removal quantity of Se(IV) by nZVI/rGO composites was calculated using the following equation (*q_e_*) [27]:

(1) $$q_{e}=\frac{\left(C_{0}-C_{e}\right)V}{m}$$

where *q_e_* (mg·g^−1^) stands for the removal quantity of the Se(IV) per unit mass of the adsorbent, *C*_0_ (mg·L^−1^) and *C_e_* (mg·L^−1^) represent the initial and equilibrium concentrations of the Se(IV), respectively, *V* (L) stands for the volume of the solution, and *m* (g) is the dry weight of the nZVI/rGO composites. The removal percentage (R) of Se(IV) was calculated using the following equation [36]:

(2) $$R=\frac{C_{0}-C_{t}}{C_{0}}\times 100\%$$

where *C*_0_ (mg·L^−1^) and *C_t_* (mg·L^−1^) represent the concentrations of initial and after time t, respectively. 

### 2.3. Response Surface Methodology

RSM is a main branch of experimental design, which is used to evaluate the effect of several factors and their interaction on the system response. This method is useful for developing and optimizing the independent variables and response, and providing the smaller number of experimental runs [27]. The Box-Behnken design (BBD) model is a standard RSM [37], which can be applied to evaluate the interactive effects of adsorption variables and optimize the removal process. Specifically, C (30–40 mg/L), initial pH (6–8), t (50–70 min) and T (20–30 °C) were selected as independent variables, and the removal efficiency of Se(IV) as the dependent variable. The experimental design involved four parameters, each at three levels, which were coded as shown in Table 1. For the evaluation of experimental data of Se (IV) removal, the response variable was fitted by the quadratic polynomial equation given below:

*Y* = *α*_0_ + *α*_1_*x*_1_ + *α*_2_*x*_2_ + *α*_3_*x*_3_ + *α*_4_*x*_4_ + *α*_12_*x*_1_*x*_2_ + *α*_13_*x*_1_*x*_3_ + *α*_14_*x*_1_*x*_4_ + *α*_23_*x*_2_*x*_3_ + *α*_24_*x*_2_*x*_4_ + *α*_34_*x*_3_*x*_4_ + *α*_11_*x*_1_^2^ + *α*_22_*x*_2_^2^ + *α*_33_*x*_3_^2^ + *α*_44_*x*_4_^2^ (3)

where *Y* stands for the removal efficiency of Se(IV) used as the dependent variable, *x*_1_*, x*_2_*, x*_3_*,* and *x*_4_ represent the independent variables, and *α*_0_*, α*_1_*, α*_2_*, α*_3_*, α*_4_*_,_ α*_12_*, α*_13_*, α*_14_*, α*_23_*, α*_24_*,* *α*_34_*, α*_11_*, α*_22_*, α*_33_ and *α*_44_ represent the model coefficients, respectively.

### 2.4. Artificial Neural Network

**Figure 1:**
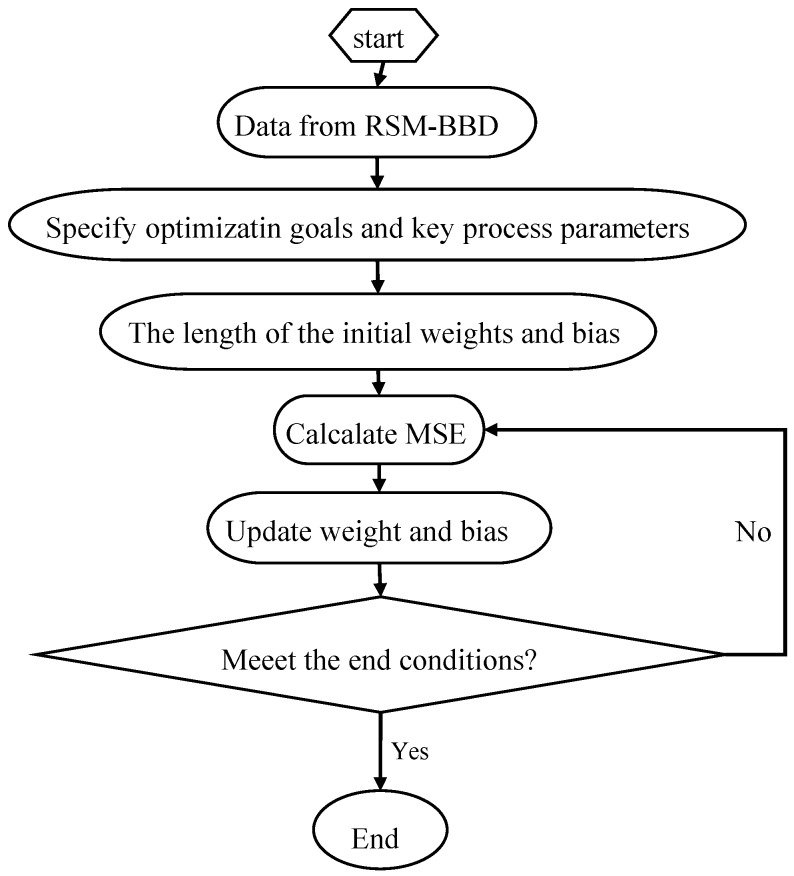
Flow diagram of neural network (ANN) training.

ANN is a good inspiration of human brain and nerve systems that are known for their superior ability to learn and classify the data, thus a feed-forward multilayer perception (MLP) ANN model was utilized in the present study with a back propagation (BP) algorithm, which contains an input layer, a hidden layer and an output layer [27]. The initial Se(IV) concentration, initial pH, operating temperature and contact time were utilized as input parameters, while the removal efficiency of Se(IV) was used as the output parameter. Among the 29 datasets generated from RSM, 24 datasets were employed to train the network and remaining 5 datasets were used for testing of the ANN model [38]. The performance of the ANN models was determined based on the criteria, such as mean squared error (*MSE*) and the correlation coefficient (*R*^2^), which can be shown as follows [39]:

(4) $$MSE=\frac{1}{N}\sum_{i=1}^{N}{\left(y_{prd,i}-y_{exp,i}\right)}^{2}$$

(5) $$R^{2}=1-\frac{\sum_{i=1}^{N}\left(y_{prd,i}-y_{exp,i}\right)}{\sum_{i=1}^{N}\left(y_{prd,i}-y_{m}\right)}$$

where *y_prd,i_* stands for the predicted value by ANN model, *y_exp,i_* represents the experimental value, *N* stands for the number of data, and *y_m_* represents the average of the experimental value. Figure 1 is a flow diagram of ANN training:

Sensitivity analysis was carried out to explore the connection weights of the trained ANN [29]. Analysis based on the magnitude of weights are based exclusively on the values stored in the static matrix of weights in order to determine the relative influence of each input variable on each one of the network outputs [40]. The equation below is proposed by Garson for this type of analysis [40,41]:

(6) $$G_{ik}=\frac{\sum_{j=1}^{n}\left(\frac{|w_{ij}|}{\sum_{r=1}^{m}\left|w_{rj}\right|}\left|w_{jk}\right|\right)}{\sum_{e=1}^{m}\left(\sum_{j=1}^{n}\left(\frac{\left|w_{ij}\right|}{\sum_{r=1}^{m}\left|w_{rj}\right|}\left|w_{jk}\right|\right)\right)}$$

where *G_ik_* stands for the percentage of influence of the dependent variable *x_i_* on the independent variable *Y_k_*, *w* represents the connection weight, *i*, *j* and *k* stand for the number of neurons in the input layer, hidden layer and output layer, respectively.

### 2.5. Genetic Algorithm

**Figure 2:**
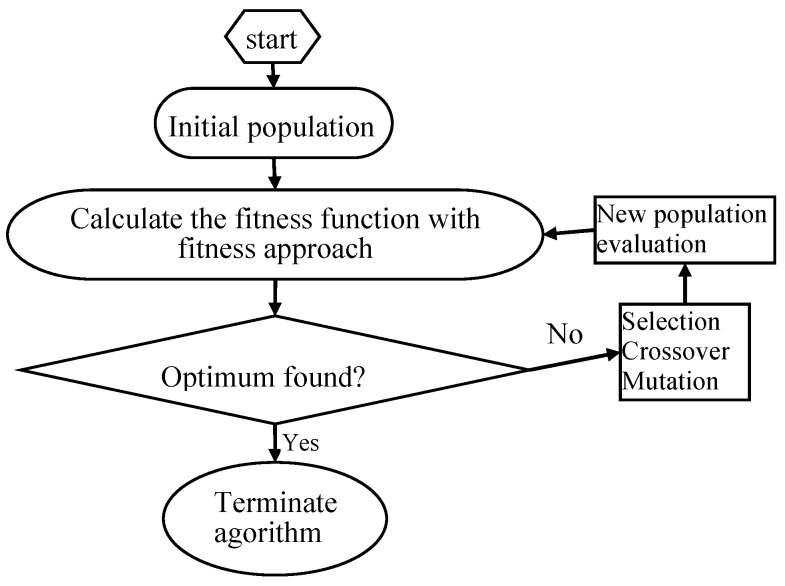
Schematic representation of the optimization rationale for genetic algorithm (GA).

GA has been proven to be a successful method for solving the optimization problems by mimicking the principle of biological evolution [42]. GA based optimization processes can be executed using trained ANN models as the fitness functions to give a global optimum solution [43], which applies crossover, mutation, and selecting operators to a population of encoded variables space. [44,45]. The values of GA-specific parameters used in the optimization technique were as follows: the number of variables = 4, maximum generation = 100, size of population = 20, crossover probability = 0.8 and mutation probability = 0.01, bounds (C: 30–40 mg/L, initial pH: 6–8, t: 50–70 min and T: 20–30 °C). The flow diagram of genetic algorithm is shown in Figure 2. Specific steps were as follows [46]: (1) initial population was chosen randomly; (2) every individual of the population was evaluated; (3) the population with the lowest MSE value (best individual) was selected for the next generation (elitism); (4) the best parents were chosen to generate the children using the GA operators; (5) the same procedure was repeated for the second generation and the algorithm was run for a defined number of cycles. (6) after finishing the GA optimization, the BP algorithm started from the solution provided by GA.

### 2.6. Particle Swarm Optimization

**Figure 3:**
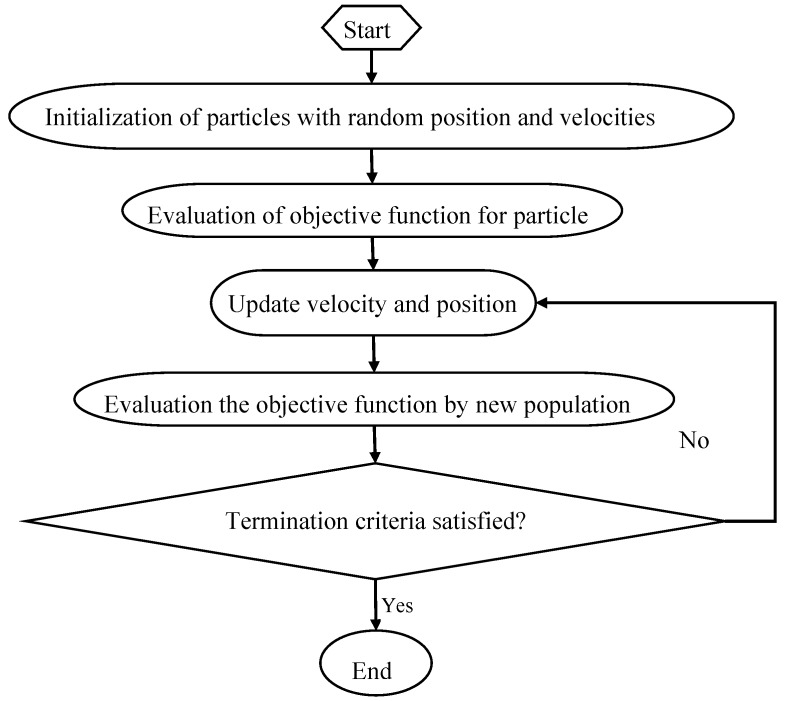
Schematic representation of the optimization rationale for for particle swarm optimization (PSO).

PSO is an evolutionary computational algorithm proposed by Kennedy and Eberhart in 1995, which is based on the simulation of natural habits of bird flocking [32]. PSO algorithm uses a population of particles that has the ability to evolve during the search for an optimal solution. The system is initialized randomly and the particles fly through the multi-dimensional search space at a certain velocity [47]. PSO computes the particles based on a fitness function and finds a particle with a good position. If a better position is obtained, the previous value will be replaced by the current value. No two particles are the same and each still learns the attributes of others that will help improve their fitness [48]. PSO consists of different control parameters, including acceleration coefficients (c1: personal learning coefficient, c2: global learning coefficient), swarm size, and inertia weight. Wrong initialization of these parameters may lead to divergent or cyclic behavior. The minimum inertia weight, maximum inertia weight, c1, c2, swarm size and maximum iteration number were set as: 0.3, 0.9, 2, 2, 20 and 50. The flow chart of PSO is shown in Figure 3:

### 2.7. Equilibrium Adsorption Studies

The equilibrium adsorption isotherm is employed to give useful information concerning properties, tendency and mechanism of adsorption of Se(IV) onto nZVI/rGO composites. The data obtained from the equilibrium adsorption study were fitted to different adsorption isotherm equations such as Langmuir, Freundlich and the Redlich Peterson models to discuss the adsorption characteristics [36]. 

Based on monolayer adsorption onto homogeneous adsorbent surface, the Langmuir isotherm is expressed as follows [39]:

(7) $$\frac{C_{e}}{q_{e}}=\frac{1}{K_{L}q_{\max}}+\frac{C_{e}}{q_{\max}}$$

where *C_e_* (mg·L^−1^) stands for the equilibrium Se(IV) concentration in the solution, *q*_max_ (mg·g^−1^) demonstrates the maximum adsorption capacity, *q*_e_ (mg·g^−1^) represents the equilibrium amount of Se(IV) adsorbed per unit mass of sorbent, *K_L_* (L·mg^−1^) stands for the Langmuir constant related to adsorption energy. The favorability of Langmuir isotherm was judged based on the dimensionless equilibrium parameter (*R_L_*) [39]:

(8) $$R_{L}=\frac{1}{1+K_{L}C_{0}}$$

where *R_L_* indicates the type of isotherm to be favorable (0 < *R_L_* < 1), irreversible (*R_L_* = 0), linear (*R*_L_ = 1) or unfavorable (*R_L_* > 1), *C*_0_ (mg·L^−1^) stands for the initial Se(IV) concentration and *K_L_* represents the Langmuir constant. 

The Freundlich isotherm is generally employed to investigate the behavior and mechanism of adsorption base on heterogeneous surface. The equation of Freundlich isotherm can be represented as below [39]:

(9) $$\ln q_{e}=\ln K_{F}+\frac{1}{n}\ln C_{e}$$

where *K_F_* [(mg·g^−1^)/(mg·L^−1^)^1/n^] stands for the Freundlich constant give useful knowledge about adsorption capacity and n is a coefficient related to adsorption intensity. 

The experimental data of Se(IV) removal were also fitted to the Redlich Peterson isotherm, which can be expressed as follows [49]:

(10) $$q_{e}=\frac{AC_{e}}{1+BC_{e}}$$

where *A* (L·g^−1^) stands for the Redlich Peterson isotherm constant, and *B* (L·mg ^(1/A)^^−1^) represents the Redlich Peterson isotherm constant. In addition, the statistical analysis was performed to investigate the validity of isotherms on the basis of two parameters, e.g., chi square test (*x*^2^) and the sum of absolute errors (*SAE*), which were evaluated using the following equations [50]:

(11) $$SAE={\sum_{i=1}^{n}\left|q_{e,\exp}-q_{e,cal}\right|}_{i}$$

(12) $$x^{2}={\sum_{i=1}^{n}\left[\frac{q_{e,\exp}-q_{e,cal}}{q_{e,cal}}\right]}_{i}$$

where *q_e,exp_* and *q*_e,cal_ stand for the experimental and calculated adsorption capacity (mg·g^−1^) and *n* represents the number of measurements. 

### 2.8. Kinetic Studies

Kinetic study was performed to investigate the removal rate that is important in design and modeling of the process. The pseudo-first-order (Equation (13), pseudo-second-order (Equation (14)) and intraparticle diffusion (Equation (15)) were used to perform the kinetic study [36,51,52].

(13) $$\ln(q_{e}-q_{t})=\ln q_{e}-k_{1}t$$

(14) $$\frac{t}{q_{t}}=\frac{1}{k_{2}q_{e}{}^{2}}+\frac{t}{q_{e}}$$

(15) $$q_{t}=k_{d}t^{0.5}+C$$

where *q_e_* (mg·g^−1^) and *q_t_* (mg·g^−1^) stands for the removal quantity of Se(IV) at equilibrium and at time *t*, *k*_1_ (1·min^−1^) represents the rate constant of pseudo-first-order removal process, *k*_2_ (g·mg^−1^ min^−1^) is the pseudo-second-order rate constant of removal, *k_d_* (mg·g^−^^1^·min^−^^0.5^) represents the intraparticle diffusion constant, and *C* as the intercept (mg·g^−^^1^·min^−^^0.5^).

### 2.9. Thermodynamic Study

Thermodynamics of Se(IV) adsorption onto nZVI/rGO was employed to evaluate the exothermic, spontaneity or endothermic nature of the removal process at the adsorbate–adsorbent interface [36]. Thermodynamic parameters, viz. Gibbs’ free energy change (*ΔG*^0^), enthalpy change (*ΔH*^0^) and entropy change (*ΔS*^0^) were investigated through the following equations:

(16) $$\ln K_{T}=\frac{\Delta S^{0}}{R}-\frac{\Delta H^{0}}{RT}$$

(17) $$\Delta G^{0}=-RT\ln K_{T}$$

(18) $$K_{T}=\frac{C_{0}}{C_{e}}$$

where *K_T_* stands for the distribution coefficient, *R* (8.314 J·mol^−1^ K^−1^) represents the gas constant, *T* (K) represents the temperature, *C*_0_ (mg·L^−1^) and *C_e_* (mg·L^−1^) represent the initial and equilibrium concentrations of Se(IV), respectively.

## 3. Results and Discussion

### 3.1. RSM Modeling

The relationship between the independent variables and response can be described by a quadratic model. The empirical relationship between the removal efficiency of Se(IV) (*Y*) and the independent variables studied (*x*_1_, *x*_2_, *x*_3_, *x*_4_) is represented as follows:

*Y* = 79.63 − 0.89*x*_1_ − 0.53*x*_2_ − 4.85*x*_3_ + 5.79*x*_4_ − 0.13*x*_1_*x*_2_ − 0.99*x*_1_*x*_3_ + 0.59*x*_1_*x*_4_ + 1.32*x*_2_*x*_3_ − 1.52*x*_2_*x*_4_ + 0.10*x*_3_*x*_4_ + 0.43 *x*_1_^2^ + 0.45*x*_2_^2^ − 0.14*x*_3_^2^ + 0.81*x*_4_^2^

The significance of quadratic regression model was tested by the correlation coefficient, F-value, *p*-value, and the corresponding results of ANOVA are presented in Table 2. The F-value (51.27) and the *p*-value (less than 0.0001) of this model implied that the model was highly significant for Se(IV) removal by nZVI/rGO. “Adequate Precision” measures the signal to noise ratio, and the ratio greater than 4.0 is considered desirable [53]. The “Adequate Precision” ratio (30.5) of this model is far greater than 4.0, indicating the presence of adequate signal for the model. The optimum predicted for the maximum removal of Se(IV) by nZVI/rGO is about 94.45%, and the corresponding optimal parameters of removal process are as follows: t = 70.00 min, initial pH = 7.97, T = 30.00 °C and C = 30 mg·L^−1^.

**Figure 4:**
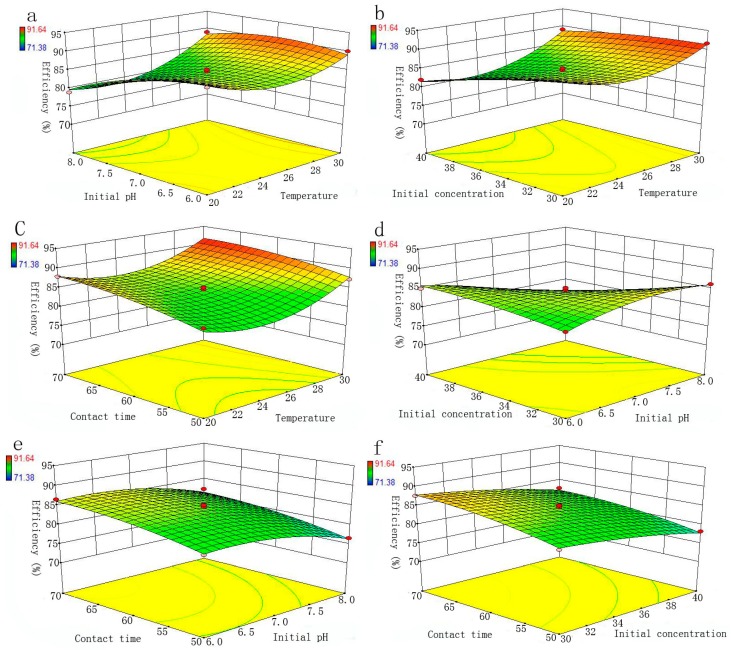
The 3-D surface plots for interactive effect of temperature and initial pH (**a**); initial Se(IV) concentration and temperature (**b**); contact time and temperature (**c**); initial pH and initial Se(IV) concentration (**d**); contact time and initial pH (**e**) and contact time and initial Se(IV) concentration (**f**); on the removal of the Se(IV) (the red dot represents the center point).

In order to further understand the effects of mutual interaction of the independent variables on the response, the 3-D surface plots are displayed in Figure 4. These plots can indicate the interactive effect of two independent variables by holding other factors at a fixed level for the removal of Se(IV) by nZVI/rGO composites. 

### 3.2. ANN Modeling

**Figure 5:**
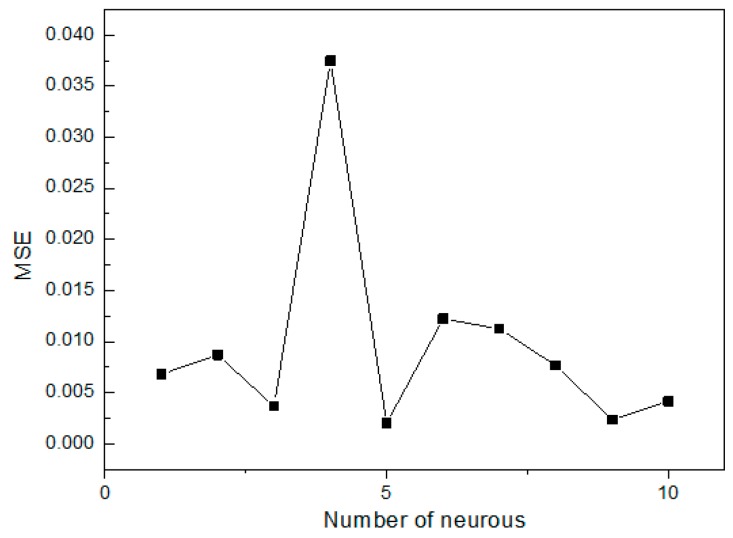
Effect of neuron number on the performance of ANN using mean squared error (*MSE*) as a criterion.

**Figure 6:**
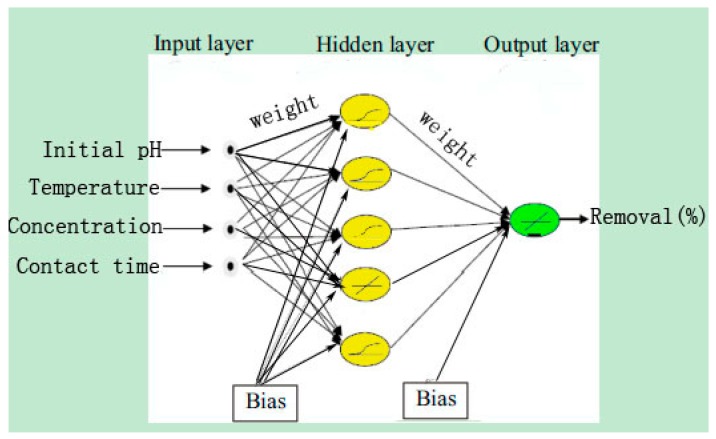
Illustration of back propagation (BP)-ANN architecture (the yellow oval represents neurons in the hidden layer; the green oval represents neurons in the output layer).

**Figure 7:**
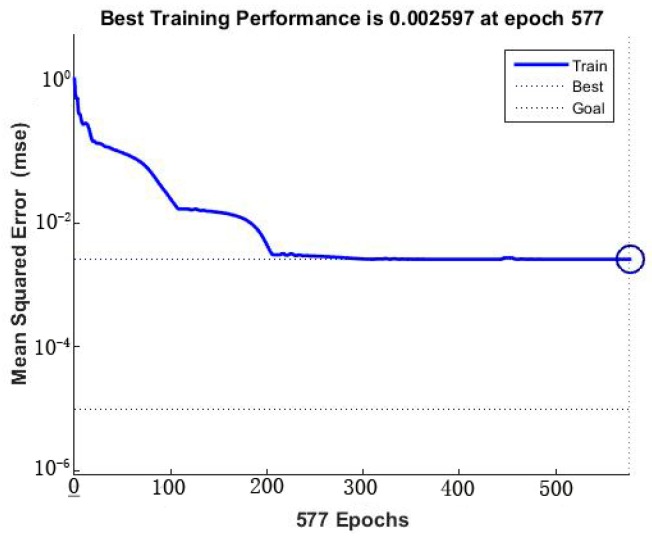
MSE vs. the number of epochs.

The optimal architecture of the ANN model was selected based on the maximum value of *R*^2^ and minimum value of the *MSE* of testing set [54]. The gradient descent with momentum and adaptive learning rate (traingdx function) was used to evaluate the ANN training [55]. The epoch was set as 2000, the learning rate was designed at 0.1, the goal was set as 1 × 10^−5^, and momentum factor was set as 0.9. The ANN training was stopped when the epoch reached 577 and the goal reached 8.01 × 10^−6^. In the network optimization, we used 1 to 10 neurons in the hidden layer. The number of 5 neurons in the hidden layer was found to be the most suitable structure for the Se(IV) removal process. (Figure 5), and the *R*^2^ and *MSE* reached the values of 0.9949 and 0.0020, respectively. Based on the approximation of MSE function, a three layered BP-ANN was used for the modeling of the removal process (Figure 6) [56]. The *MSE* against the number of epochs for the optimal ANN model is shown in Figure 7, which demonstrated that the training was stopped after about 577 epochs. Sensitivity analysis demonstrates that contact time appears to be the most influential variable for Se(IV) removal, followed by initial pH, operating temperature and initial concentration (Table 3).

### 3.3. GA Optimization

**Figure 8:**
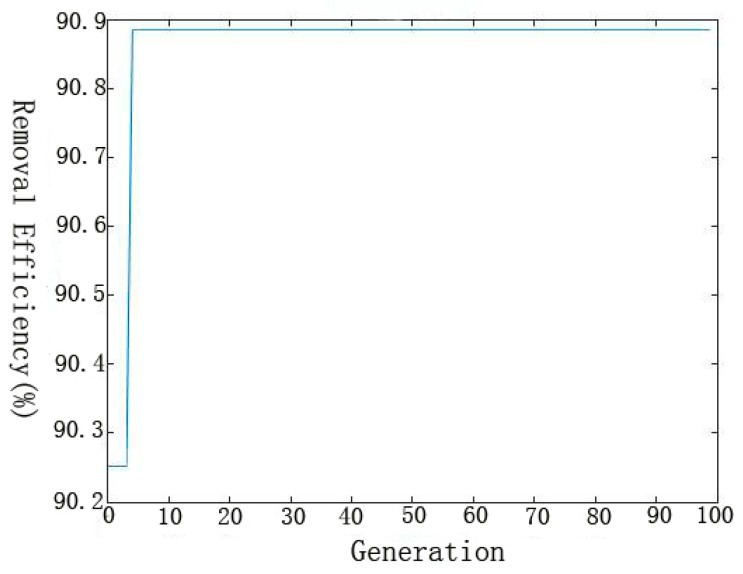
Evolvement of fitness with 100 generations.

GA was employed to optimize the weights and thresholds of ANN for an objective to maximize the removal efficiency of Se(IV) [42,45]. Optimum conditions were selected after the evaluation of GA for about 4 generations to achieve a good removal efficiency of Se(IV) (Figure 8). The removal efficiency was 90.89% under the optimized conditions from ANN-GA model (T = 29.65 °C, initial pH = 6.55, C = 36.13 mg/L and t = 64.22 min). The removal efficiency from the confirmatory experiment was 88.01%, and the residual error between the predicted and experimental values was 2.88%, confirming that ANN-GA model is feasible for modeling and optimizing the Se(IV) removal by the nZVI/rGO composites.

### 3.4. PSO Optimization

**Figure 9:**
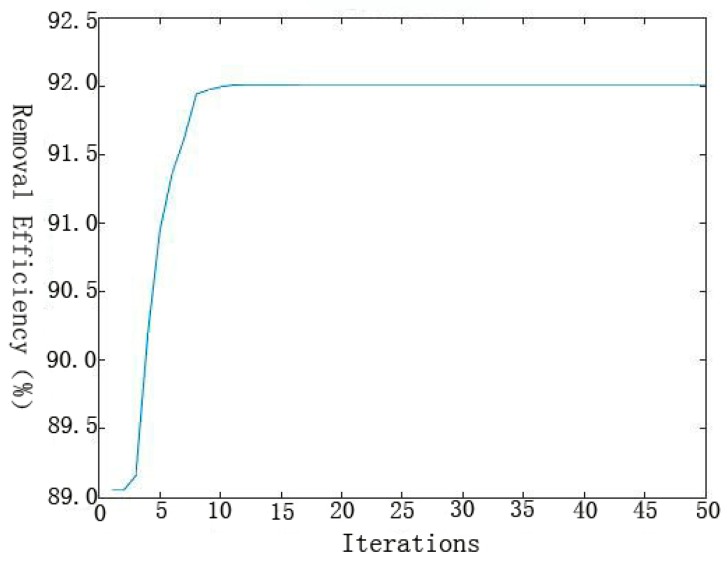
The maximum removal efficiency against iterations.

PSO was employed to search the space of the developed ANN model and find the optimum input values for the maximum removal [32,46]. of Se(IV) removal efficiency. Optimum conditions were determined after the evaluation of PSO for about 12 iterations to achieve a good removal efficiency of Se(IV) (Figure 9). The optimized process conditions are as follows: t = 60.00 min, initial pH = 6.78, T = 23.74 °C and = 30 mg·L^−1^. The removal efficiency predicted by the ANN-PSO model was 92.02% in comparison with 87.39% from the confirmatory experiment carried out under the optimized conditions from ANN-PSO model. The residual error (4.63%) shows that ANN-PSO model is an effective tool for the Se(IV) removal process since the predicted value is close to the results of confirmatory experiment.

### 3.5. Comparison of Different Models

In the present study, the results obtained from the models (RSM model, ANN-GA model and ANN-PSO model) are validated and compared by confirmatory experiments carried out under the predicted optimal operating conditions (Table 4). The ANN model possesses a good accuracy with the higher *R*^2^ value (0.9949) than that of RSM (0.9809), furthermore, the residual error of ANN-GA model (2.88%) is lower than that of the ANN-PSO model (4.63%) and the RSM model (5.56%). Therefore, the ANN-GA methodology may present a satisfactory choice for Se(IV) removal from aqueous solutions by nZVI/rGO composites.

### 3.6. Adsorption Isotherms

**Figure 10:**
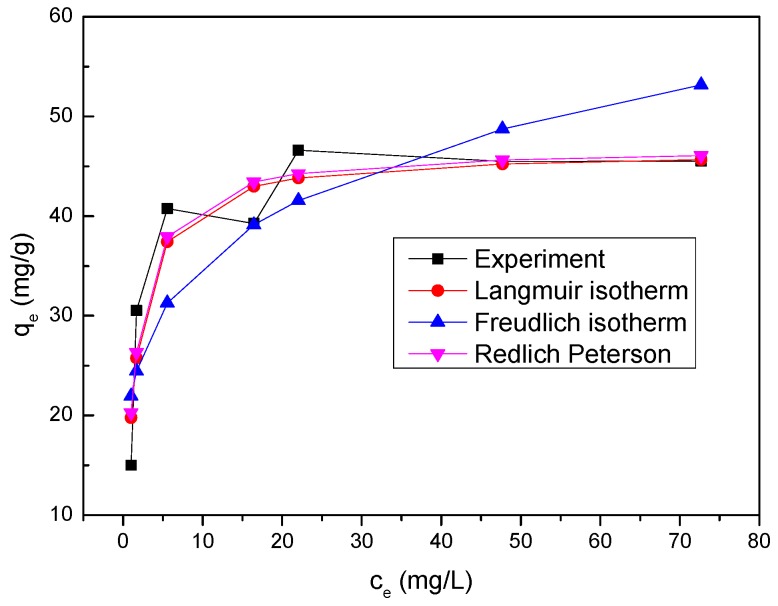
Adsorption isotherm for Se(IV) onto nZVI/rGO composites (nZVI/rGO dosage = 30 mg; initial pH = 8.0; t = 1 h; T = 25 °C).

The values of *R*^2^ and other parameters of the three models are summarized in Table 5, from which it can be found that the adsorption of Se(IV) on nZVI/rGO composites could be more suitably described by the Langmuir model than by the Freundlich model or by the Redlich Peterson model due to higher *R*^2^ value and lower values of *x*^2^, *SAE*. This showed that the adsorption process for the nZVI/rGO composites was controlled by the monolayer adsorption on a homogeneous surface. The removal quantity of the nZVI/rGO composites is significantly higher than that of other materials (Table 6). The calculated *R_L_* values for the adsorption of Se(IV) onto the nZVI/rGO composites at different initial concentrations are listed in Table 7. All *R_L_* values calculated were between 0 and 1, thus this adsorption process is favorable. For the Freundlich isotherm model, the value of n was 4.4897. It is generally stated that the value of *n* in the range 2–10, 1–2 and less than 1 represents a good, moderately difficult and poor adsorption, respectively [57]. The value of n suggests the satisfactory adsorption of Se(IV) onto the nZVI/rGO composites. The isotherm plots for Se(IV) adsorption are shown in Figure 10, which indicates that the removal quantity calculated by the Langmuir model is in general more compatible with the experimental data than that calculated by the Freundlich model and the Redlich Peterson model.

### 3.7. Kinetics Study

**Figure 11:**
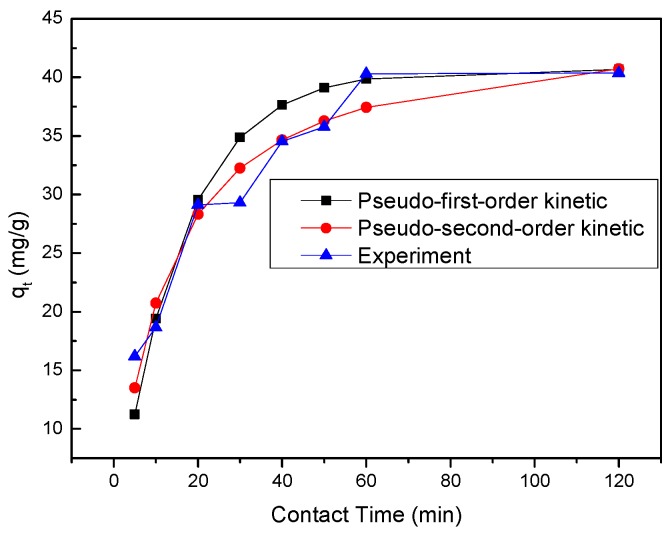
Time dependent study of Se(IV) removal by nZVI/rGO composites (initial pH = 8.0; nZVI/rGO dosage = 30 mg; T = 25 °C; C = 30 mg/L).

The parameters for the three kinetic models are given in Table 8. The *R*^2^ value of the pseudo-second-order kinetic model (0.9938) was significantly higher than that of the pseudo-first-order kinetic model (0.6134) and the intraparticle diffusion model (0.8590). This indicates that the removal process is best described by the pseudo-second-order kinetic model. The removal process of Se(IV) by the nZVI/rGO composites reached the equilibrium within 60 min (Figure 11), and most of *q_e_* computed by using the pseudo-second-order kinetic model were compatible with the experimental data.

### 3.8. Thermodynamics Studies

The thermodynamic values of *ΔG*^0^, *ΔH*^0^ and *ΔS*^0^ obtained for the removal process of Se(IV) by nZVI/rGO composites are summarized in Table 9. The enthalpy change is related to the chemical bonding, therefore, the positive value of the enthalpy of adsorption *ΔH*^0^ obtained in this study confirms the endothermic nature of the adsorption process [63]. The values of *ΔG*^0^ at various temperatures were negative, indicating the spontaneous nature of the adsorption process [64]. The value of *ΔS*^0^ is related to the order or the geometry of the adsorbed SeO_3_^2^^−^. The positive value of ΔS^0^ suggests an increase in disorder and randomness at the solid-solution interface during the adsorption process [63]. In addition, the positive values of *ΔH*^0^ and *ΔS*^0^ demonstrated that the Se(IV) adsorption onto the nZVI/rGO composites is a entropy-driven process [65].

### 3.9. Se(IV) Removal Mechanisms

**Figure 12:**
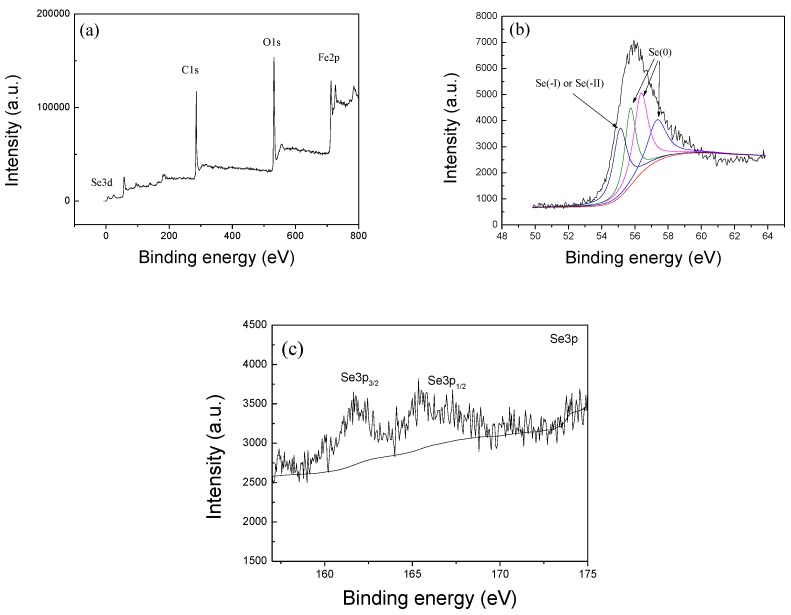
Wide-scan XPS survey of nZVI/rGO-Se(IV) (**a**); high-resolution spectrum of Se 3d for nZVI/rGO-Se(IV) (**b**); the XPS spectra of Se 3p for nZVI/rGO-Se(IV) (**c**).

The removal process of heavy metal ions from wastewater is generally controlled by various mechanisms, such as adsorption, reduction and co-precipitation. To further understand the removal process of Se(IV), its mechanism was studied in this study. For investigating the interaction mechanisms between Se(IV) and the nZVI/rGO composites, the nZVI/rGO composites (nZVI/rGO-Se(IV)) after the Se(IV) removal was analyzed with XPS (Figure 12).

The wide-scan XPS spectrum of the nZVI/rGO composites after the Se(IV) removal indicated that the surface of the nZVI/rGO composites consists mainly of carbon, oxygen, iron and selenium (Figure 12aThe peaks of Se(0), Se(−I) and Se(−II) are shown in the spectrum of Se 3d for nZVI/rGO-Se(IV) (Figure 12b) [66], and the peak at 54.8 eV is attributed to Se(−I) or Se(−II) [67]. Furthermore, the peaks of Se 3p are observed on XPS spectra for nZVI/rGO-Se(IV) (Figure 12c [68]. These results showed that the removal process of Se(IV) by nZVI/rGO was mainly controlled by adsorption and reduction. 

## 4. Conclusions

In this study, artificial intelligence tools (i.e., ANN, GA and PSO) were applied to investigate the effects of different operating conditions (initial pH, temperature, contact time and initial concentration) on the removal of Se(IV) by the nZVI/rGO composites from wastewater. The results showed that the ANN model offered a better prediction than the RSM model with a higher value of *R*^2^ (0.9949). The ANN model gave a more precise prediction of the experimental data with a satisfactory value of *R*^2^ (0.9949) than the RSM model. The result predicted by the ANN-GA model is better than that by the ANN-PSO model and by the RSM model, which indicated that the ANN-GA model was an effective tool for modeling and optimizing the Se(IV) removal process. Furthermore, the experimental data were best described by the Langmuir isotherm and the adsorption process is favorable (0 < R_L_ ˂ 1). The removal kinetics of Se(IV) by the nZVI/rGO composites was successfully fitted to the pseudo-second-order kinetic model and the removal quantity calculated is generally more compatible with the experimental data. Thermodynamic parameters obtained demonstrated that the Se(IV) removal process is endothermic and spontaneous in nature. The Se 3d and 3p peaks found in XPS spectra for nZVI/rGO-Se(IV) composites demonstrated that the removal of Se(IV) by nZVI/rGO was mainly through the adsorption and reduction mechanisms. Future work will focus on the modeling and optimization of the complex removal processes with the aid of more advanced AI techniques. In addition, the permeable reactive barrier (PRB) system will be used to investigate the heavy metals removal from wastewater.

## Figures and Tables

**Table 1:** The experimental factors and levels for the Box–Behnken design.

Factors	Name	Units	Range and Levels (Coded)		
			−1	0	1
*x*_1_	Temperature	°C	20	25	30
*x*_2_	initial pH	-	6	7	8
*x*_3_	Initial Se(IV) concentration	mg·L^−^^1^	30	35	40
*x*_4_	Time	min	50	60	70

**Table 2:** The results of ANOVA for the second-order polynomial equation.

Source	Sum of Squares	df	Mean Square	F-Value	*p*-Value	Remarks
Model	565.46	14.00	40.39	51.27	<0.0001	significant
*x*_1_	56.64	1.00	56.64	71.90	<0.0001	
*x*_2_	77.32	1.00	77.32	98.15	<0.0001	
*x*_3_	87.10	1.00	87.10	110.57	<0.0001	
*x*_4_	63.57	1.00	63.57	80.70	<0.0001	
*x*_1_ *x*_2_	17.98	1.00	17.98	22.82	0.0003	
*x*_1_ *x*_3_	6.89	1.00	6.89	8.75	0.0104	
*x*_1_ *x*_4_	0.49	1.00	0.49	0.62	0.4434	
*x*_2_ *x*_3_	71.40	1.00	71.40	90.64	<0.0001	
*x*_2_ *x*_4_	0.08	1.00	0.08	0.11	0.7487	
*x*_3_ *x*_4_	0.12	1.00	0.12	0.15	0.7074	
*x*_1_^2^	112.94	1.00	112.94	143.37	<0.0001	
*x*_2_^2^	26.50	1.00	26.50	33.64	<0.0001	
*x*_3_^2^	3.27	1.00	3.27	4.15	0.0610	
*x*_4_^2^	6.53	1.00	6.53	8.29	0.0121	

*R*^2^ = 0.9809, Adequate precision = 30.520.

**Table 3:** Variables importance analysis of input for the output in ANN model.

Input Variables	Percentage (%)	Order
Operating temperature	28.58	3
Initial pH	30.49	2
Initial concentration	4.50	4
Contact time	36.43	1

**Table 4:** The optimized process parameters for Se(IV) removal by nZVI/rGO composites using different methods.

Process Parameters	Optimization	
	ANN-GA	ANN-PSO
Operating temperature (°C)	29.65 °C	23.74 °C
Initial pH	6.55	6.78
Initial Se(IV) concentration (mg/L)	36.13 mg/L	30 mg/L
Contact time (min)	64.22 min	60.00 min
Removal efficiency of model (%)	90.89%	92.02%
Experimental verification values (%)	88.01%	87.39%
Average values of absolute errors (%)	2.88	4.63
*R*^2^	0.9949	
*MSE*	0.0020	

**Table 5:** Parameters of isotherm models for the adsorption of Se(IV) onto the nZVI/rGO composites.

Isotherms	Parameters	Values of Parameters	Error Function	
			*x* ^2^	*SAE*
Langmuir	*K_L_* (L·mg^−^^1^)	0.7414	0.0127	
	*q_max_* (mg·g^−^^1^)	46.5116		19.8041
	*R* ^2^	0.9986		
Freundlich	*K_F_* (mg·g^−^^1^)	21.9705	0.1498	
	*n*	4.8497		38.6090
	*R* ^2^	0.6904		
Redlich Peterson	*A*	35.8093	0.0845	
	*B*	0.7635		38.6090
	*R* ^2^	0.8818		

**Table 6:** Removal quantity of various adsorbents for Se(IV).

Materials	*q_max_* (mg/g)	Reference
tropical soil	0.15	[58]
Fe(III)/Cr(III) hydroxide	15.63	[59]
Mg/Fe Hydrotalcite-Like-Compound	4.70	[60]
Aluminium oxide coated sand	1.08	[61]
Fe-GAC	2.58	[62]
nZVI/rGO nanocomposites	46.51	Present work

**Table 7:** The values of R_L_ for the adsorption of Se(IV) by the nZVI/rGO composites.

Initial Concentration (mg/L)	*R_L_* Value
10	0.1188
20	0.0632
30	0.0430
40	0.0326
50	0.0263
75	0.0177
100	0.0133

**Table 8:** Kinetic parameters for removal of the Se(IV) by the nZVI/rGO composites.

Model	Parameters	Value of Parameters
pseudo-first-order	*k*_1_ (1·min^−^^1^)	0.0648
	*R* ^2^	0.6134
	*q_e_*	40.7056
pseudo-second-order	*k*_2_ (g·mg^−^^1^min^−^^1^)	0.0018
	*R* ^2^	0.9938
	*q_e_*	44.6400
Intraparticle diffusion	*k*_3_ (mg·g^−^^1^ min^−^^1/2^)	2.7259
	*B* (mg·g^−^^1^)	14.1790
	*R* ^2^	0.8590

**Table 9:** Thermodynamic parameters for Se(IV) removal by nZVI/rGO composites.

ΔH^0^ (kJ·mol^−1^)	ΔS^0^ (J·mol^−1^ K^−1^)	ΔG^0^ (kJ/mol)		
9.0531	40.7386	293 K	303 K	313 K
		−5.6658	−7.1402	−9.4507

## References

[1] Liang L., Yang W., Guan X., Li J., Xu Z., Wu J., Huang Y., Zhang X. (2013). Kinetics and mechanisms of pH-dependent selenite removal by zero valent iron. Water Res..

[2] Liang L., Guan X., Huang Y., Ma J., Sun X., Qiao J., Zhou G. (2015). Efficient selenate removal by zero-valent iron in the presence of weak magnetic field. Sep. Purif. Technol..

[3] Kuroda M., Notaguchi E., Sato A., Yoshioka M., Hasegawa A., Kagami T., Narita T., Yamashita M., Sei K., Soda S. (2011). Characterization of pseudomonas stutzeri NT-I capable of removing soluble selenium from the aqueous phase under aerobic conditions. J. Biosci. Bioeng..

[4] Kagami T., Narita T., Kuroda M., Notaguchi E., Yamashita M., Sei K., Soda S., Ike M. (2013). Effective selenium volatilization under aerobic conditions and recovery from the aqueous phase by pseudomonas stutzeri NT-I. Water Res..

[5] Fu F., Lu J., Cheng Z., Tang B. (2016). Removal of selenite by zero-valent iron combined with ultrasound: Se (IV) concentration changes, se (VI) generation, and reaction mechanism. Ultrason. Sonochem..

[6] Albert M., Demesmay C., Rocca J.L. (1995). Analysis of organic and non-organic arsenious or selenious compounds by capillary electrophoresis. Anal. Bioanal. Chem..

[7] Nováková E., Linhart O., Červený V., Rychlovský P., Hraníček J. (2017). Flow injection determination of Se in dietary supplements using TiO_2_ mediated uv-photochemical volatile species generation. Spectrochim. Acta Part B At. Spectrosc..

[8] Li J., Chen C., Zhu K., Wang X. (2016). Nanoscale zero-valent iron particles modified on reduced graphene oxides using a plasma technique for Cd (II) removal. J. Taiwan Inst. Chem. Eng..

[9] Sun Y., Ding C., Cheng W., Wang X. (2014). Simultaneous adsorption and reduction of U (VI) on reduced graphene oxide-supported nanoscale zerovalent iron. J. Hazard. Mater..

[10] Kerkez D.V., Tomašević D.D., Kozma G., Bečelić-Tomin M.R., Prica M.D., Rončević S.D., Kukovecz Á., Dalmacija B.D., Kónya Z. (2014). Three different clay-supported nanoscale zero-valent iron materials for industrial azo dye degradation: A comparative study. J. Taiwan Inst. Chem. Eng..

[11] Chen Z., Wang T., Jin X., Chen Z., Megharaj M., Naidu R. (2013). Multifunctional kaolinite-supported nanoscale zero-valent iron used for the adsorption and degradation of crystal violet in aqueous solution. J. Colloid Interface Sci..

[12] Wang C., Luo H., Zhang Z., Wu Y., Zhang J., Chen S. (2014). Removal of as (III) and as (V) from aqueous solutions using nanoscale zero valent iron-reduced graphite oxide modified composites. J. Hazard. Mater..

[13] Li L., Hu J., Shi X., Fan M., Luo J., Wei X. (2016). Nanoscale zero-valent metals: A review of synthesis, characterization, and applications to environmental remediation. Environ. Sci. Pollut. Res..

[14] Liu Y., Phenrat T., Lowry G.V. (2007). Effect of TCE concentration and dissolved groundwater solutes on NZVI-promoted TCE dechlorination and H_2_ evolution. Environ. Sci. Technol..

[15] Yan S., Hua B., Bao Z., Yang J., Liu C., Deng B. (2010). Uranium (VI) removal by nanoscale zerovalent iron in anoxic batch systems. Environ. Sci. Technol..

[16] Lv X., Xu J., Jiang G., Xu X. (2011). Removal of chromium (VI) from wastewater by nanoscale zero-valent iron particles supported on multiwalled carbon nanotubes. Chemosphere.

[17] Zheng T., Zhan J., He J., Day C., Lu Y., Mcpherson G.L., Piringer G., John V.T. (2008). Reactivity characteristics of nanoscale zerovalent iron–silica composites for trichloroethylene remediation. Environ. Sci. Technol..

[18] Macosko C. (2010). Graphene/polymer nanocomposites. Macromolecules.

[19] Xia G., Tan Y., Chen X., Fang F., Sun D., Li X., Guo Z., Yu X. (2017). Oxygen-free layer-by-layer assembly of lithiated composites on graphene for advanced hydrogen storage. Adv. Sci..

[20] Stankovich S., Dikin D.A., Piner R.D., Kohlhaas K.A., Kleinhammes A., Jia Y., Wu Y., Nguyen S.B.T., Ruoff R.S. (2007). Synthesis of graphene-based nanosheets via chemical reduction of exfoliated graphite oxide. Carbon.

[21] Feng W., Long P., Feng Y., Li Y. (2016). Two-dimensional fluorinated graphene: Synthesis, structures, properties and applications. Adv. Sci..

[22] Wang X., Yu S., Jin J., Wang H., Alharbi N.S., Alsaedi A., Hayat T., Wang X. (2016). Application of graphene oxides and graphene oxide-based nanomaterials in radionuclide removal from aqueous solutions. Sci. Bull..

[23] Dong Z., Wang D., Liu X., Pei X., Chen L., Jin J. (2014). Bio-inspired surface-functionalization of graphene oxide for the adsorption of organic dyes and heavy metal ions with a superhigh capacity. J. Mater. Chem. A.

[24] Yuan Y., Zhang G., Li Y., Zhang G., Zhang F., Fan X. (2013). Poly(amidoamine) modified graphene oxide as an efficient adsorbent for heavy metal ions. Polym. Chem..

[25] Romanchuk A.Y., Slesarev A.S., Kalmykov S.N., Kosynkin D.V., Tour J.M. (2013). Graphene oxide for effective radionuclide removal. Phys. Chem. Chem. Phys..

[26] Chen Y., Chen L., Bai H., Li L. (2013). Graphene oxide-chitosan composite hydrogels as broad-spectrum adsorbents for water purification. J. Mater. Chem. A.

[27] Shojaeimehr T., Rahimpour F., Khadivi M.A., Sadeghi M. (2014). A modeling study by response surface methodology (RSM) and artificial neural network (ANN) on Cu^2+^ adsorption optimization using light expended clay aggregate (LECA). J. Ind. Eng. Chem..

[28] Gao Z., Zhang D., Ge Y. (2010). Design optimization of a spatial six degree-of-freedom parallel manipulator based on artificial intelligence approaches. Robot. Comput. Integr. Manuf..

[29] Zhang Y., Pan B. (2014). Modeling batch and column phosphate removal by hydrated ferric oxide-based nanocomposite using response surface methodology and artificial neural network. Chem. Eng. J..

[30] Ghaedi M., Shojaeipour E., Ghaedi A.M., Sahraei R. (2015). Isotherm and kinetics study of malachite green adsorption onto copper nanowires loaded on activated carbon: Artificial neural network modeling and genetic algorithm optimization. Spectrochim. Acta Part A Mol. Biomol. Spectrosc..

[31] Jiang B., Zhang F., Sun Y., Zhou X., Dong J., Zhang L. (2014). Modeling and optimization for curing of polymer flooding using an artificial neural network and a genetic algorithm. J. Taiwan Inst. Chem. Eng..

[32] Khajeh M., Kaykhaii M., Sharafi A. (2013). Application of pso-artificial neural network and response surface methodology for removal of methylene blue using silver nanoparticles from water samples. J. Ind. Eng. Chem..

[33] Chandra V., Park J., Chun Y., Lee J.W., Hwang I.C., Kim K.S. (2010). Water-dispersible magnetite-reduced graphene oxide composites for arsenic removal. ACS Nano.

[34] Fan M., Li T., Hu J., Cao R., Wu Q., Wei X., Li L., Shi X., Ruan W. (2016). Synthesis and characterization of reduced graphene oxide-supported nanoscale zero-valent iron (nZVI/rGO) composites used for Pb (II) removal. Materials.

[35] Fan M., Li T., Hu J., Cao R., Wei X., Shi X., Ruan W. (2017). Artificial neural network modeling and genetic algorithm optimization for cadmium removal from aqueous solutions by reduced graphene oxide-supported nanoscale zero-valent iron (nZVI/rGO) composites. Materials.

[36] Dil E.A., Ghaedi M., Ghaedi A., Asfaram A., Jamshidi M., Purkait M.K. (2016). Application of artificial neural network and response surface methodology for the removal of crystal violet by zinc oxide nanorods loaded on activate carbon: Kinetics and equilibrium study. J. Taiwan Inst. Chem. Eng..

[37] Kumar R., Singh R., Kumar N., Bishnoi K., Bishnoi N.R. (2009). Response surface methodology approach for optimization of biosorption process for removal of Cr (VI), Ni (II) and Zn (II) ions by immobilized bacterial biomass sp. Bacillus brevis. Chem. Eng. J..

[38] Shi X., Ruan W., Hu J., Fan M., Cao R., Wei X. (2017). Optimizing the removal of rhodamine B in aqueous solutions by reduced graphene oxide-supported nanoscale zerovalent iron (nZVI/rGO) using an artificial neural network-genetic algorithm (ANN-GA). Nanomaterials.

[39] Ghaedi M., Daneshfar A., Ahmadi A., Momeni M.S. (2015). Artificial neural network-genetic algorithm based optimization for the adsorption of phenol red (PR) onto gold and titanium dioxide nanoparticles loaded on activated carbon. J. Ind. Eng. Chem..

[40] Montaño J.J., Palmer A. (2003). Numeric sensitivity analysis applied to feedforward neural networks. Neural Comput. Appl..

[41] Kasiri M.B., Aleboyeh H., Aleboyeh A. (2008). Modeling and optimization of heterogeneous photo-fenton process with response surface methodology and artificial neural networks. Environ. Sci. Technol..

[42] Karimi H., Ghaedi M. (2014). Application of artificial neural network and genetic algorithm to modeling and optimization of removal of methylene blue using activated carbon. J. Ind. Eng. Chem..

[43] Zafar M., Kumar S., Kumar S., Dhiman A.K. (2012). Artificial intelligence based modeling and optimization of poly(3-hydroxybutyrate-co-3-hydroxyvalerate) production process by using azohydromonas lata MTCC 2311 from cane molasses supplemented with volatile fatty acids: A genetic algorithm paradigm. Bioresour. Technol..

[44] Yasin Y., Ahmad F.B.H., Ghaffari-Moghaddam M., Khajeh M. (2014). Application of a hybrid artificial neural network–genetic algorithm approach to optimize the lead ions removal from aqueous solutions using intercalated tartrate-mg–al layered double hydroxides. Environ. Nanotechnol. Monit. Manag..

[45] Bagheri M., Mirbagheri S.A., Bagheri Z., Kamarkhani A.M. (2015). Modeling and optimization of activated sludge bulking for a real wastewater treatment plant using hybrid artificial neural networks-genetic algorithm approach. Process Saf. Environ. Prot..

[46] Karimi H., Yousefi F. (2012). Application of artificial neural network–genetic algorithm (ANN–GA) to correlation of density in nanofluids. Fluid Phase Equilib..

[47] Dhanarajan G., Mandal M., Sen R. (2014). A combined artificial neural network modeling–particle swarm optimization strategy for improved production of marine bacterial lipopeptide from food waste. Biochem. Eng. J..

[48] Das G., Pattnaik P.K., Padhy S.K. (2014). Artificial neural network trained by particle swarm optimization for non-linear channel equalization. Expert Syst. Appl..

[49] Kumar K.V., Porkodi K., Rocha F. (2008). Comparison of various error functions in predicting the optimum isotherm by linear and non-linear regression analysis for the sorption of basic red 9 by activated carbon. J. Hazard. Mater..

[50] Rahman N., Haseen U. (2014). Equilibrium modeling, kinetic, and thermodynamic studies on adsorption of pb (II) by a hybrid inorganic–organic material: Polyacrylamide zirconium (IV) iodate. Ind. Eng. Chem. Res..

[51] Yuh-Shan H. (2004). Citation review of lagergren kinetic rate equation on adsorption reactions. Scientometrics.

[52] Ho Y.S., Mckay G. (1998). Sorption of dye from aqueous solution by peat. Chem. Eng. J..

[53] Han C., Pu H., Li H., Deng L., Huang S., He S., Luo Y. (2013). The optimization of As (V) removal over mesoporous alumina by using response surface methodology and adsorption mechanism. J. Hazard. Mater..

[54] Ghaedi M., Ghaedi A.M., Abdi F., Roosta M., Sahraei R., Daneshfar A. (2014). Principal component analysis-artificial neural network and genetic algorithm optimization for removal of reactive orange 12 by copper sulfide nanoparticles-activated carbon. J. Ind. Eng. Chem..

[55] Ghanbary F., Modirshahla N., Khosravi M. (2012). Synthesis of TiO_2_, nanoparticles in different thermal conditions and modeling its photocatalytic activity with artificial neural network. J. Environ. Sci..

[56] Aleboyeh A., Kasiri M.B., Olya M.E., Aleboyeh H. (2008). Prediction of azo dye decolorization by UV/H_2_O_2_ using artificial neural networks. Dyes Pigment..

[57] Yao Y., Miao S., Liu S., Ma L.P., Sun H., Wang S. (2012). Synthesis, characterization, and adsorption properties of magnetic Fe_3_O_4_@graphene nanocomposite. Chem. Eng. J..

[58] Goh K.H., Lim T.T. (2004). Geochemistry of inorganic arsenic and selenium in a tropical soil: Effect of reaction time, pH, and competitive anions on arsenic and selenium adsorption. Chemosphere.

[59] Namasivayam C., Prathap K. (2006). Removal of selenite using ‘waste’ Fe (III)/Cr (III) hydroxide: Adsorption kinetics and isotherms. Toxicol. Environ. Chem..

[60] Das J., Das D., Dash G.P., Parida K.M. (2002). Studies on mg/fe hydrotalcite-like-compound (HTLc) I. Removal of inorganic selenite (SeO_3_^2−^) from aqueous medium. J. Colloid Interface Sci..

[61] Kuan W.H., Lo S.L., Wang M.K., Lin C.F. (1998). Removal of Se (IV) and Se (VI) from water by aluminum-oxide-coated sand. Water Res..

[62] Zhang N., Lin L.S., Gang D. (2008). Adsorptive selenite removal from water using iron-coated GAC adsorbents. Water Res..

[63] Ismaiel A.A., Aroua M.K., Yusoff R. (2013). Palm shell activated carbon impregnated with task-specific ionic-liquids as a novel adsorbent for the removal of mercury from contaminated water. Chem. Eng. J..

[64] Pan S., Shen H., Xu Q., Luo J., Hu M. (2012). Surface mercapto engineered magnetic Fe_3_O_4_ nanoadsorbent for the removal of mercury from aqueous solutions. J. Colloid Interface Sci..

[65] Silva S.M., Sampaio K.A., Ceriani R., Verhé R., Stevens C., Greyt W.D., Meirelles A.J.A. (2013). Adsorption of carotenes and phosphorus from palm oil onto acid activated bleaching earth: Equilibrium, kinetics and thermodynamics. J. Food Eng..

[66] Canava B., Vigneron J., Etcheberry A., Guillemoles J.F., Lincot D. (2002). High resolution XPS studies of Se chemistry of a Cu(In, Ga)Se_2_ surface. Appl. Surf. Sci..

[67] Jung B., Safan A., Batchelor B., Abdel-Wahab A. (2016). Spectroscopic study of se (IV) removal from water by reductive precipitation using sulfide. Chemosphere.

[68] Sun W., Pan W., Wang F., Xu N. (2015). Removal of Se (IV) and Se (VI) by MFe_2_O_4_ nanoparticles from aqueous solution. Chem. Eng. J..

